# Genetic Spectrum of Familial Hypercholesterolemia in Russian North-West Registry: Focus on Correlation Between Clinical and Genetic Diagnosis

**DOI:** 10.3390/jcdd13080384

**Published:** 2026-08-12

**Authors:** Maria I. Krivosheina, Viktoria V. Bakaleiko, Olga V. Reutova, Polina S. Sokolnikova, Anna A. Kostareva, Asiiat S. Alieva

**Affiliations:** 1 Institute of Molecular Biology and Genetics, Almazov National Medical Research Centre, 2 Akkuratova, Saint-Petersburg 197341, Russia; krivosheina_mi@almazovcentre.ru (M.I.K.); reutova_ov@almazovcentre.ru (O.V.R.); sokolnikova_ps@almazovcentre.ru (P.S.S.); anna.kostareva@ki.se (A.A.K.); 2 Department of Faculty Therapy, Almazov National Medical Research Centre, Saint-Petersburg 197341, Russia; alieva_as@almazovcentre.ru

**Keywords:** LDL-C, familial hypercholesterolemia, LDLR, NGS

## Abstract

Genetic analysis of patients with lipid metabolism disorders is becoming increasingly prevalent in clinical practice. In particular, researchers are directing considerable attention toward studying familial hypercholesterolemia (FH). Despite its high population frequency (heterozygous FH occurs in approximately 1 in 250–300 individuals, with even higher prevalence reported in some regions of the world), the heterogeneity of this condition presents major obstacles to its diagnosis. Owing to the advancement of molecular methods and laboratory technologies, it has become possible to establish the genetic basis of metabolic disorders. In the present study, using next-generation sequencing (NGS), a cohort of 261 patients with a confirmed clinical diagnosis of familial hypercholesterolemia was investigated. Among the 82 variants identified in FH-associated genes (*LDLR*, *APOB*, *PCSK9*, *LDLRAP1*), the most prevalent was the *LDLR* NP_000518.3:p.Leu401His variant, which was detected in 21 patients. The second most frequent variant in our cohort (19 patients) was *APOB* NP_000433.2:p.Arg3527Gln. Overall, 56.1% of patients with a DLCN score of 3–5 (possible FH) carried pathogenic variants, demonstrating the insufficient sensitivity of clinical criteria alone. The presence of early coronary heart disease in 22.73% of genotype-negative patients, together with the considerable number of individuals displaying a pronounced clinical FH phenotype despite negative genetic testing, underscores the importance of further exploring the genetic architecture of FH across different populations.

## 1. Introduction

Familial hypercholesterolemia (FH) is an inherited disorder of cholesterol metabolism that results in elevated plasma levels of low-density lipoprotein cholesterol (LDL-C). In healthy individuals, an LDL cholesterol level exceeding 3.0 mmol/L is considered elevated, whereas a plasma LDL concentration above 5.0 mmol/L is critical for all patient groups and warrants further investigation to rule out familial hypercholesterolemia and other genetic causes. The pathogenesis of atherosclerosis in FH is driven by the lifelong exposure of the vascular wall to extremely high levels of LDL-C, resulting from impaired receptor-mediated LDL catabolism [1]. Atherogenesis is initiated by the subendothelial retention of apolipoprotein B-containing lipoproteins, followed by vascular wall injury, monocyte recruitment, transformation of macrophages into foam cells, and the subsequent formation of atherosclerotic plaques [2]. The presence of genetically confirmed pathogenic or likely pathogenic variants in genes providing LDL-C formation and utilization (predominantly in the *LDLR*, *APOB*, or *PCSK9* genes) is associated with an exceptionally high risk of premature atherosclerotic cardiovascular disease (ASCVD) [3], including coronary heart disease (CHD), which remains the leading cause of death worldwide, accounting for approximately 16% of all global mortality [4].

Heterozygous familial hypercholesterolemia (HeFH) is one of the most common genetic disorders, with an estimated prevalence of approximately 1 in 300 individuals worldwide [5]. It is characterized by the presence of a pathogenic or likely pathogenic variants in one of the causative genes (*LDLR*, *APOB*, *PCSK9*) in the monoallelic state. When the causative variants (in homozygous or compound heterozygous state) are detected in both alleles of the gene, diagnosis of homozygous FH (HoFH) is established. Consequently, HoFH is considerably rarer, with an estimated prevalence ranging from 1 in 250,000 to 1 in 360,000 individuals, and is associated with a more severe phenotype and earlier manifestation [5].

Variants in the *LDLR* gene are the most common cause of FH, accounting for 85–90% of genetically confirmed cases [6]. Pathogenic variants in *LDLR* diminish receptor function, thereby compromising LDL-C uptake and clearance [7]. Pathogenic variants in the apolipoprotein B (*APOB*) gene, which impair LDL binding to the LDL receptor, and gain-of-function variants in the proprotein convertase subtilisin/kexin type 9 (*PCSK9*) gene, which enhance LDL receptor degradation, are less common and account for approximately 5–15% and 1% of FH cases, respectively. Autosomal recessive FH caused by homozygous pathogenic variants in the LDL receptor adaptor protein 1 (*LDLRAP1*) gene, while being extremely rare, should also be considered [6]. The p.Leu167del variant in *APOE*, inherited as an autosomal dominant trait, is reported to explain 1% to 2% of FH-phenotype hypercholesterolemia cases in certain national cohorts [5,8].

Despite its high prevalence, familial hypercholesterolemia remains largely underdiagnosed and, consequently, undertreated [9]. Left untreated, patients develop premature CHD, typically manifesting before the age of 55 in men and 60 in women. While early detection and adequate management can significantly mitigate this risk [10], many individuals with FH still fail to achieve recommended LDL-C targets. Because these patients face up to a 13-fold increased risk of CHD, prompt diagnosis and the early initiation of appropriate therapy are critical for preventing ASCVD and reducing cumulative LDL-C exposure over a lifetime [11].

Currently, the Dutch Lipid Clinic Network (DLCN), Simon Broome, and Make Early Diagnoses to Prevent Early Deaths (MEDPED) criteria are widely used for the initial screening and clinical diagnosis of familial hypercholesterolemia (FH) [5]. These scoring systems enable the clinical identification of FH even in the absence of genetic testing, thereby facilitating the timely initiation of lipid-lowering therapy. However, the European Atherosclerosis Society (EAS) consensus statement recommends genetic testing to identify pathogenic or likely pathogenic variants in causative genes for patients with a DLCN score > 5 [11]. Despite advances in molecular genetics, no definitive pathogenic variant is identified in 10–40% of patients who meet the clinical criteria for FH. In many instances, this diagnostic gap is driven by a high burden of variants of uncertain significance (VUS), whose clinical interpretation remains challenging. The ClinVar database currently registers over 5000 variants associated with FH, and various literature sources indicate that 40–60% of these are classified as VUS. Resolving the pathogenicity of these molecular defects requires specialized in vitro functional assays to evaluate the low-density lipoprotein receptor’s capacity to bind and clear LDL particles from the plasma. Because these studies demand complex comparative analyses between variant-expressing cells and wild-type controls, they remain largely restricted to specialized research facilities. Consequently, less than 15% of *LDLR* variants have undergone rigorous functional characterization [12].

Beyond unresolved monogenic defects, the absence of a causative variants in clinically diagnosed patients reflects the complex and heterogeneous etiology of severe hypercholesterolemia. A subset of patients with a clinically established diagnosis of familial hypercholesterolemia are carriers of variants in genes responsible for other lipid metabolism disorders. These include sitosterolemia (a rare hereditary condition characterized by elevated plasma levels of plant sterols or non-cholesterol sterols) [13], dysbetalipoproteinemia (a mixed hyperlipidemia characterized by elevated plasma cholesterol and triglyceride levels) [14], and others. The clinical phenotype can be driven by polygenic inheritance—where the cumulative impact of multiple small-effect LDL-raising variants mimics a monogenic disorder—or by the involvement of novel, yet-to-be-discovered disease-associated genes [15,16]. Crucially, the clinical presentation of FH is often significantly influenced by elevated levels of lipoprotein(a) [17]. Because the cholesterol mass of the Lp(a) particle is captured as LDL-C in standard laboratory lipid panels, high Lp(a) concentrations can artificially inflate the calculated LDL-C level, leading to an overestimation of the DLCN score and a pseudo-FH diagnosis. Moreover, elevated Lp(a) is an independent, genetically determined risk factor for atherosclerotic cardiovascular disease, meaning its presence not only mimics the FH lipid profile but synergistically amplifies the overall cardiovascular risk, underscoring the necessity of routine Lp(a) measurement in this patient population.

**Figure 1:**
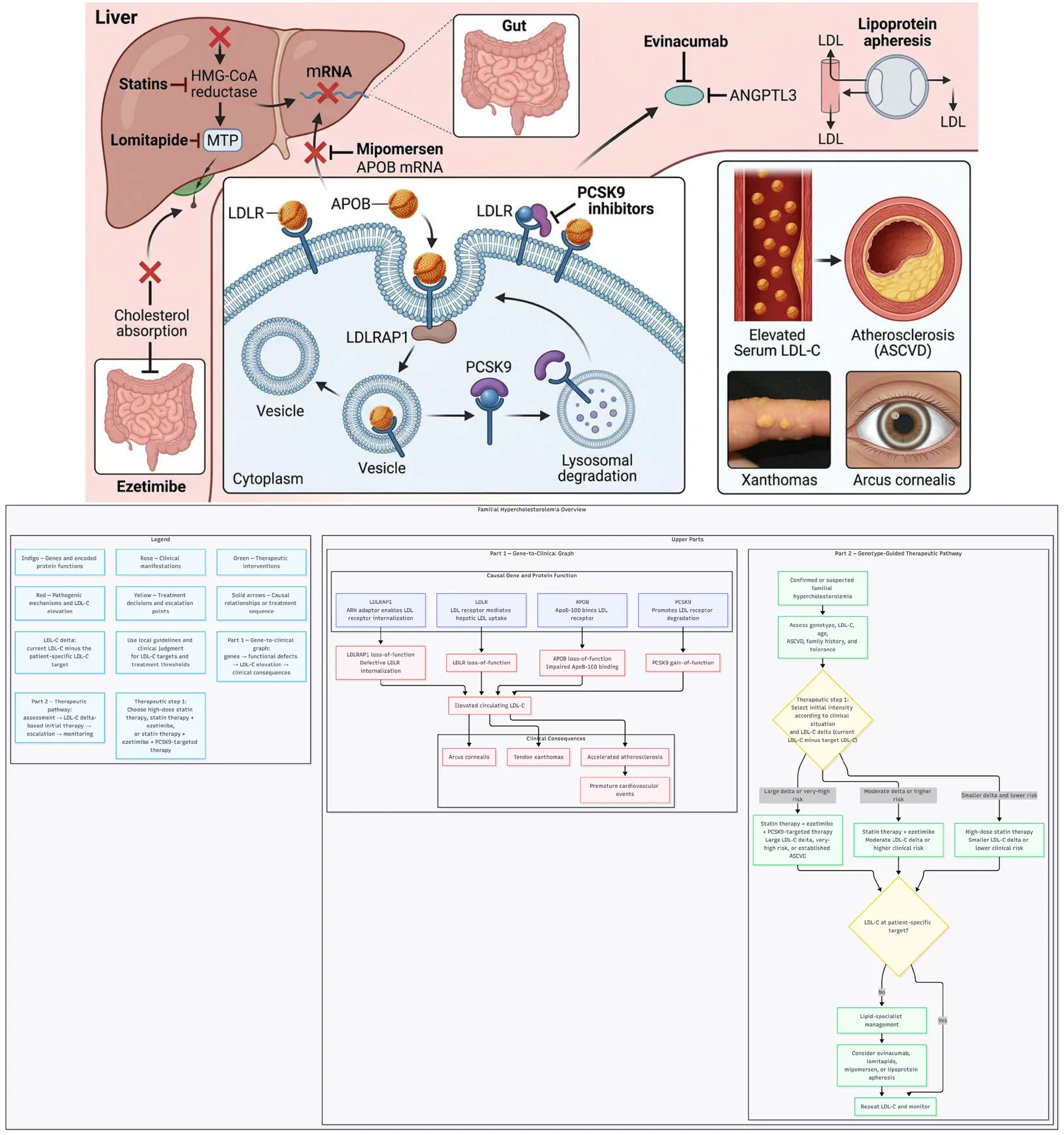
The pathomechanism, clinical features, and therapeutic approaches to familial hypercholesterolemia.

In this study, we comprehensively investigated the genetic etiology of FH in a cohort of Russian patients presenting with an FH phenotype according to DLCN criteria. We aimed to expand the spectrum of variants in known disease-associated genes using multiple molecular genetic approaches. The study cohort was identified through a population-based FH screening program with an additional inclusion of genetic testing. By improving our understanding of genotype–phenotype relationships in FH, this work may contribute to the optimization of treatment strategies and ultimately improve patient outcomes. Figure 1 illustrates the pathomechanism, clinical manifestations, and therapeutic approaches to familial hypercholesterolemia as a function of the patient’s genetic diagnosis.

## 2. Materials and Methods

### 2.1. Study Population

The study included 261 patients (209 probands and 52 relatives) from the Almazov National Medical Research Centre, aged 18 years and older, with a confirmed diagnosis of familial hypercholesterolemia. The diagnosis was established using the clinical criteria of the Dutch Lipid Clinic Network (DLCN).

Inclusion criteria were as follows: a total DLCN score of >5 for adult probands and >4 for adult relatives. Atherosclerosis was diagnosed in patients based on the identification of intima-media thickening via ultrasonography. Cardiovascular events were defined as a history of myocardial infarction, stroke, or revascularization.

### 2.2. DNA Extraction and Sequencing

Peripheral blood samples from patients were collected into vacuum tubes containing EDTA and biobanked at −20 °C until analysis. DNA was extracted from the blood using the FlexiGene DNA Kit (Qiagen, Hilden, Germany). Library preparation was performed using the SureSelect XT Library Prep Kit (Agilent Technologies, Santa Clara, CA, USA).

Molecular genetic analysis of probands was performed using next-generation sequencing (NGS) with targeted enrichment according to the Illumina technology (PE 2 × 150 bp), employing a panel of genes and polymorphisms (*ABCG5*, *ABCG8*, *ANGPTL3*, *APOA1*, *APOA4*, *APOA5*, *APOB*, *APOC2*, *APOC3*, *APOE*, *CETP*, *GPD1*, *GPIHBP1*, *LCAT*, *LDLR*, *LDLRAP1*, *LIPA*, *LMF1*, *LPL*, *LRP6*, *PCSK9*, *SAR1B*, *SLC25A40*, *STAP1*, *TNFRSF1B*, *USF1*, rs4149056, rs2032582, rs3798220, rs12975366). The panel was developed at the Institute of Molecular Biology and Genetics, Almazov National Medical Research Centre. Genetic testing of patients’ relatives and confirmation of NGS results were performed using Sanger sequencing.

Genetic analysis and interpretation were examined across all genes of the panel. Patients with pathogenic and likely pathogenic variants in genes responsible for the development of FH (*LDLR*, *APOB*, *PCSK9*, *LDLRAP1*) were considered as genotype-positive. Patients with variants of uncertain significance in genes responsible for the development of FH or single variant in *LDLRAP1* gene, with variants in phenocopy genes (*ABCG5*, *ABCG8*, *LIPA*) or in FH candidate genes (*APOE*, *STAP1*), and in other genes associated with lipid metabolism disorders (*APOA1*, *APOA4*, *APOC3*, *TNFRSF1B*, *CETP*, *GPIHBP1*, *LMF1*, *LPL*, and *LRP6*) were considered as patients with disputed variants (Table 1). Henceforth, all the aforementioned genes, with the exception of the four major genes that represent a proven cause of FH, will be referred to in the text as “other genes”.

### 2.3. Data Analysis

The data obtained following primary analysis of the libraries on the Illumina MiSeq platform were processed using the bioinformatics tools BWA-MEM (http://www.eposters.net/pdfs/snppet-a-fast-and-sensitive-algorithm-for-variant-detection-and-confirmation-from-targeted.pdf, accessed on 6 August 2026), Picard (v.1.128; http://broadinstitute.github.io, accessed on 6 August 2026), GATK (v.3.3–0; http://www.broadinstitute.org/, accessed on 6 August 2026), and Annovar (Philadelphia, PA, USA).

The pathogenicity of the identified genetic variants was determined in accordance with the classification system of the American College of Medical Genetics and Genomics (ACMG) [26] and a special ClinGen criteria for *LDLR* gene [27]. Population allele frequencies of the variants were obtained from the Genome Aggregation Database (gnomAD; https://gnomad.broadinstitute.org/, accessed on 6 August 2026). To predict the functional effect of the identified variants, we employed the following in silico prediction tools: SIFT (v6.2.1; a-star.edu.sg, accessed on 6 August 2026), PolyPhen-2 (v2.2.3; http://genetics.bwh.harvard.edu/pph2/, accessed on 6 August 2026), and CADD (Combined Annotation Dependent Depletion; https://cadd.gs.washington.edu/, accessed on 6 August 2026).

### 2.4. Statistical Analysis

Statistical analysis of the data was performed using MS Excel and Jamovi v2.5. Normality of the sample distribution was assessed using the Anderson–Darling, D’Agostino–Pearson, Kolmogorov–Smirnov, and Shapiro–Wilk tests. For non-normally distributed quantitative variables, the median and interquartile range were calculated; for normally distributed variables, the mean, standard deviation, and Student’s *t*-test were used. The presence of a statistically significant difference among three or more groups of quantitative data was determined using the Kruskal–Wallis test, while comparisons between two groups were performed using the non-parametric Mann–Whitney U test. Categorical variables were presented as absolute and relative frequencies. Fisher’s exact test was used to establish statistical associations between two groups. A *p*-value of <0.05 was considered statistically significant.

## 3. Results

### 3.1. Clinical Characteristics

In the present study, 261 patients aged 18 to 82 years (mean age 42 ± 13 years) were investigated including 108 males (41.38%) and 153 females (58.62%). The mean age at diagnosis was 38 ± 11 years for males and 46 ± 14 years for females (*p* < 0.0001). A comparison of clinical and laboratory parameters of patients from different age groups and patients with possible, probable, and definite FH is presented in Supplementary Tables S1 and S2.

The median highest LDL-C level measured in life was 7.57 (6.63–8.87) mmol/L. The median LDL-C level at the time of patient enrolment in the study was 4.83 (3.18–6.46) mmol/L. The clinical and laboratory data of the patients are presented in Table 2.

### 3.2. Genetic Analysis

**Figure 2:**
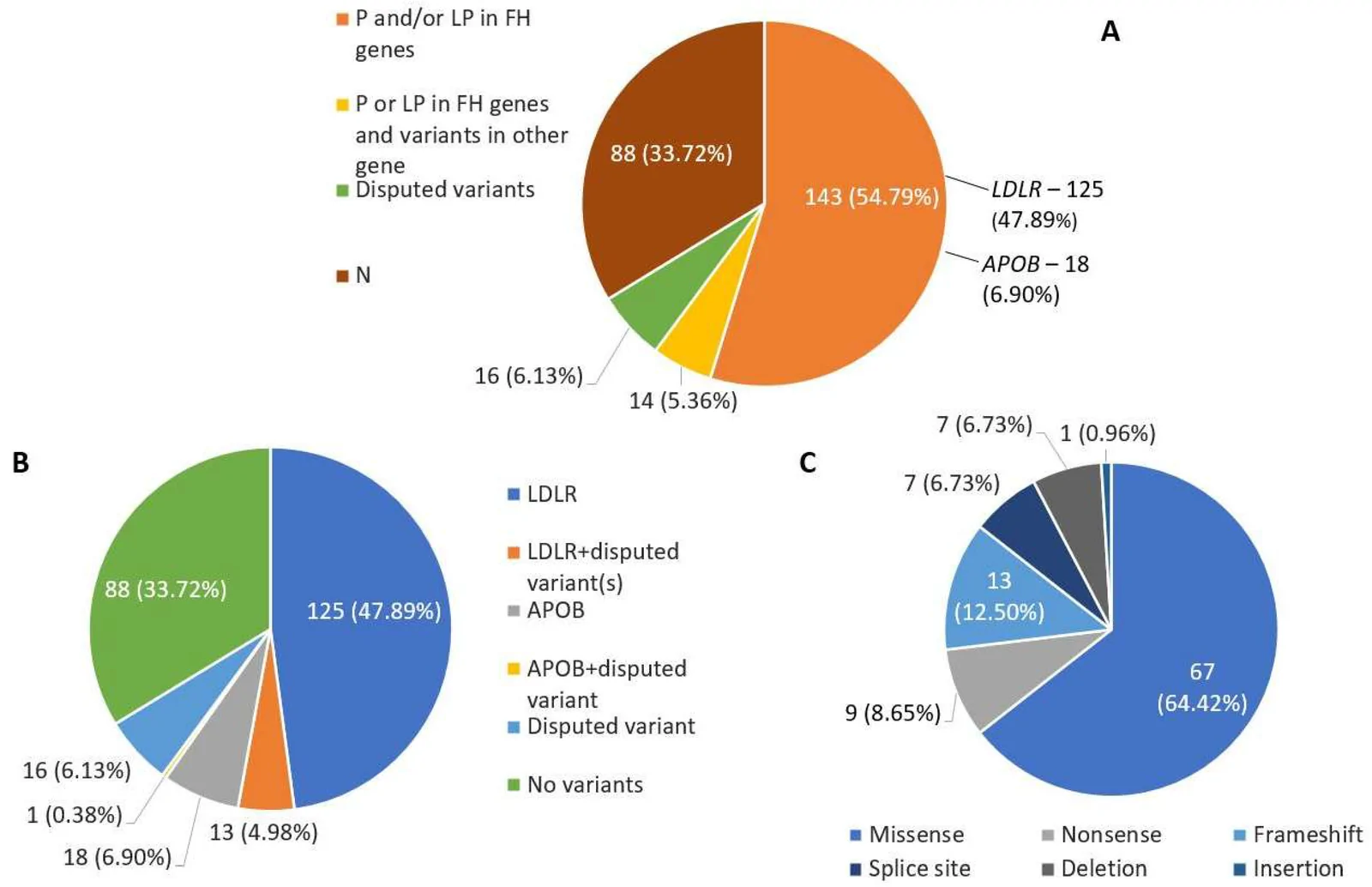
(**A**) Distribution of the patients according to variant pathogenicity; (**B**) Distribution of the variants according to the genes (in cases where two or more variants were identified, the group identification was made according to pathogenic or likely pathogenic variant). (**C**) Distribution of identified variants by type of DNA alteration. Abbreviations: FH, familial hypercholesterolemia; P, pathogenic; LP, likely pathogenic; N, no variants.

Of the 261 patients, pathogenic and likely pathogenic variants in familial hypercholesterolemia genes (*LDLR*, *APOB*, *PCSK9*, *LDLRAP1*) were detected in 157 (60.2%), of them in 54,8% the variants were detected only in FH-associated genes (Figure 2A, Table 2 and Supplementary Table S3) and in 5,36% in addition to mutations in FH genes variants in other lipid metabolism genes were identified (*ABCG5*, *ABCG8*, *APOE*, *LIPA*, *STAP1*, *APOA1*, *APOA4*, *TNFRSF1B*, *CETP*, *GPIHBP1*, *LMF1*, *LPL*, *LRP6*). In 16 patients (6.13%) only the variants of unknown clinical significance were found; these patients were considered as patients with disputed variants. No causal or disputed variants were detected in 88 patients (33.72%). The largest number of patients—140 (53.64%)—were carriers of variants in the *LDLR* gene. Variants in the *APOB* gene were detected in 20 (7.66%) patients, in the *PCSK9* gene in 2 (0.77%) patients, and in the *LDLRAP1* gene in 2 (0.77%) patients (Figure 2B and Supplementary Tables S4 and S5). All patients were heterozygous for all identified variants.

As a result, a total of 104 variants were identified: 81 (77.88%) in FH genes and 23 (22.12%) in other lipid metabolism genes. Figure 2B presents the number of identified variants in each FH gene and in other genes: the majority of variants—76 (73.08%)—were detected in the *LDLR* gene. All identified variants, along with their population frequency, CADD pathogenicity score (a variant score above 20 increases the likelihood of a pathogenic effect on the protein) and the number of observed cases is presented in Supplementary Tables S4 and S5.

Among all variants identified in our study, 48 (46.15%) were classified as pathogenic, 32 (30.77%) as likely pathogenic, and 24 (23.08%) as variants of uncertain significance, according to ACMG recommendations and their modifications for *LDLR* gene [27]. We were able to identify several common variants frequently repeated in several patients. The most prevalent pathogenic variant, detected in 21 patients, was a variant in the *LDLR* gene (chr19:g.11113293T>A; NM_000527.5:c.1202T>A; NP_000518.3:p.Leu401His; rs121908038) followed by the second most prevalent variant (19 patients) in *APOB* gene (chr2:g.21006288C>T; NM_000384.3:c.10580G>A; NP_000433.2:p.Arg3527Gln; rs5742904). The third most prevalent (12 patients) was a variant in the *LDLR* gene (chr19:g.11116928G>A; NM_000527.5:c.1775G>A; NP_000518.3:p.Gly592Glu; rs137929307) (Supplementary Tables S4 and S5).

Missense variants were the most frequent among all identified variants, comprising 64.42% (Figure 2C). Of note, 34.61% of the variants comprised nonsense, frameshift, or other types of loss-of-function variants, mainly detected in the *LDLR* gene.

To identify differences in clinical manifestations among patients with familial hypercholesterolemia according to genetic diagnosis, we analyzed two subgroups of patients in accordance with genotype-positive and genotype-negative status. The first group comprised 157 patients (60.15%) carrying pathogenic and likely pathogenic variants in FH genes, while the second group included 88 patients (33.72%) without any variants. Patients with variants of uncertain significance in FH genes (*n* = 4) and patients with pathogenic, likely pathogenic variants, or variants of uncertain significance in other lipid metabolism genes (*n* = 12) were not included in current analysis. Table 3 and Supplementary Table S3 summarize the main clinical parameters attributed to each group.

A statistically significant difference between the patient groups was identified for the following parameters:

- Age at disease diagnosis (*p* = 0.0017)—in carriers of variants in FH genes, the clinical diagnosis was established at a significantly younger age on average;
- Both LDL-C level at the time of enrolment (*p* = 0.0265) and the highest LDL-C level measured in life (*p* = 0.0005) were higher in variant carriers;
- Triglyceride’s level (*p* = 0.0239) and HDL-C level (*p* = 0.0337) at the time of enrolment were significantly higher in patients without variants;
- Presence of tendon xanthomas (*p* = 0.0028)—this major clinical feature of FH was more frequently observed in variant carriers;
- Cardiovascular events in relatives were more frequently reported (*p* = 0.0029) in patients without variants.

Based on the number of points scored on the DLCN scale, all patients were divided into three groups:

- Possible FH—3–5 points;
- Probable FH—6–8 points;
- Definite FH—≥9 points.

A comparison of the clinical and laboratory parameters of patients with possible, probable, and definite FH is presented in Supplementary Table S2.

**Figure 3:**
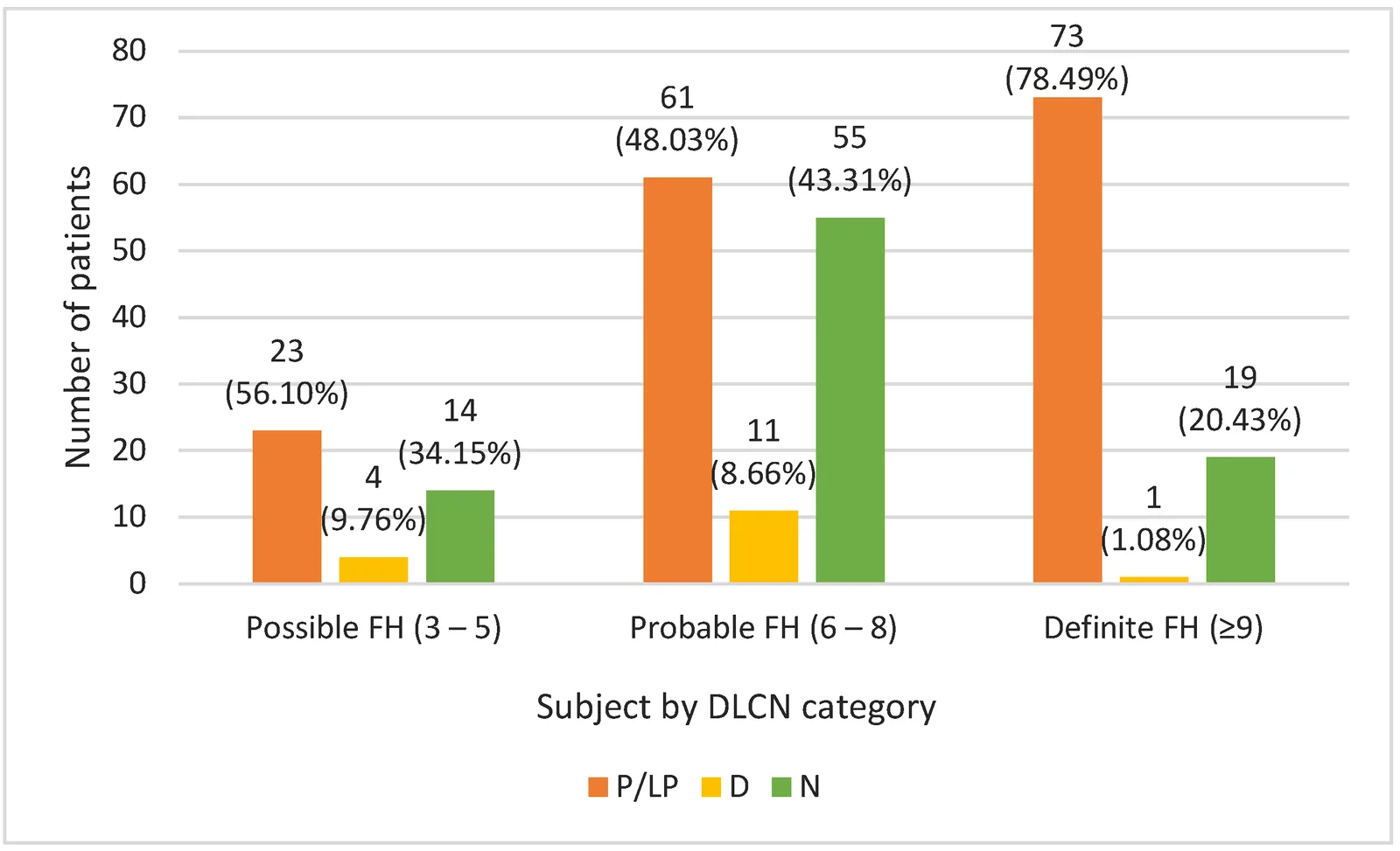
Variants in FH genes according to DLCN score; Abbreviations: FH, familial hypercholesterolemia; P, pathogenic; LP, likely pathogenic; D, disputed variants (variant of uncertain significance in all genes, pathogenic, and likely pathogenic variants in other genes); N, no variants; DLCN, Dutch Lipid Clinic Network.

Figure 3 presents the number of patients with pathogenic, likely pathogenic, and variants of uncertain clinical significance across all genes studied, as well as the number of patients without variants, in the three groups stratified by DLCN score. Of note, 43.31% of patients with a DLCN score of 6–9 and 20.43% of patients with a score ≥ 9 were genotype-negative, which in total contributes 33.72% of the “score over 6” group. If counted together with disputed variants, the group of gene-elusive patients with a DLCN score over 6 comprises 39.85%.

At the time of enrolment in the database, 118 out of 261 patients (45.21%) were not receiving lipid-lowering therapy. Table 4 presents data comparing LDL-C levels between all patients and untreated patients according to genetic diagnosis. No statistically significant difference in LDL-C levels was observed only among patients with variants of uncertain clinical significance. In all other cases, LDL-C levels were higher in untreated patients compared to the levels observed across all patients. A comparison of clinical and laboratory parameters between treated and untreated patients at the time of database enrolment is shown in Supplementary Table S6.

## 4. Discussion

In this study, we performed a comprehensive analysis of genetic, clinical, and biochemical characteristics in a cohort of patients with a FH phenotype. The primary objectives were to determine the detection rate of causative variants in lipid metabolism genes in patients with clinically proved diagnosis of FH, to evaluate the effectiveness of current clinical diagnostic approaches in relation to genetics and to assess the impact of the causative genetic background on the clinical phenotype. Understanding FH genetics in distinct local cohorts is crucial, given that variants previously reported as VUS may be reclassified as pathogenic in a specific population.

In the cohort of 261 adult patients, the proportion of genotype-positive individuals was (60.2%) being expectedly highest in the younger adult groups compared to patients aged 45–64 years and those older than 65 years. Accordingly, patients with genetically confirmed FH (carriers of pathogenic or likely pathogenic variants) had an earlier manifestation compared to genetically negative patients, suggesting earlier clinical manifestation of FH among genetically confirmed cases (*p* = 0.0037). This pattern supports the well-established notion that early manifestation of hypercholesterolemia is more likely to be explained by a monogenic cause. In older patients, a greater contribution of polygenic factors or secondary causes of dyslipidemia may attenuate the association with classic FH gene variants.

The mean maximum LDL-C level among genetically positive adult patients was higher compared to genetically negative individuals, but in contrast, lipoprotein(a) concentrations did not differ significantly between the groups, supporting the notion that this cardiovascular risk factor is largely independent of major FH-causing variants. At study enrolment, only 45.21% of patients were receiving lipid-lowering therapy but even upon therapy most patients had not achieved recommended LDL-C targets at the time of inclusion. The largest proportion of treated patients belonged to the 45–64-year age group (*n* = 60, 62.5%). Lipid-lowering as a combination of statins and ezetimibe was administered less to 1/3 pf the patients and only 10 (3.83%) patients received PCSK9 inhibitors, reflecting the limited use of advanced lipid-lowering therapies due to the lack of subsidized drug coverage for patients in primary prevention settings in the Russian Federation.

Among genotype-positive patients, the majority carried variants in *LDLR* (140 individuals, 53.64%), consistent with previous reports showing that pathogenic *LDLR* variants account for up to 90% of FH cases worldwide still with a substantial phenotypic variability reported among individuals carrying the same *LDLR* variant and complex genotype–phenotype correlations [28]. The most prevalent pathogenic variant, detected in 21 patients, was a variant in the *LDLR* gene. The pathogenic variant rs121908038 (c.1202T>A, p.Leu401His) in the *LDLR* gene is a clinically significant cause of familial hypercholesterolemia. This variant is a frequent finding among patients with FH phenotype in Russian cohorts [29,30]. At the molecular level, the substitution is located in the epidermal growth factor (EGF) precursor homology domain, resulting in the replacement of a hydrophobic leucine residue with a bulky, polar histidine [31]. This conformational alteration destabilizes the local folding of the receptor, leading to a combined class 2 and class 5 molecular defect [31]. Primarily, the aberrant structure prevents proper maturation and intracellular transport of the protein from the endoplasmic reticulum to the plasma membrane, initiating its premature degradation. Secondarily, in the fraction of receptors that successfully reach the hepatocyte surface, the mechanism of pH-dependent dissociation from the low-density lipoprotein in acidic early endosomes is disrupted. Consequently, the blockade of ligand release impairs the receptor recycling process, leading to its lysosomal degradation, severe depletion of the membrane receptor pool, and the clinical manifestation of the disease [31].

Variants in other FH-associated genes were less common accounting to less than 10% of the cases. The analysis revealed that the vast majority of cardiovascular incidents were associated with variants in the *LDLR* gene, with cardiovascular events registered in 22 individuals in this subgroup (14.0% of the entire cohort). In comparison, among patients with variants in the *APOB* gene, similar events were observed in only four individuals (2.5%). Notably, the single recorded case (0.6%) of a cardiovascular event in a patient carrying a *PCSK9* variant occurred in the setting of a combined genetic defect; this individual concurrently harbored a pathogenic variant in the *APOB* gene. This observed distribution demonstrates that *LDLR* variants are associated with the highest number of cardiovascular complications in the studied sample. These findings support the concept that pathogenic variants in the *LDLR* gene exhibit the most pronounced atherogenic potential and determine the most severe clinical phenotype of the disease compared to other monogenic causes of FH. This is attributable to the fact that patients carrying *LDLR* variants exhibit higher LDL-C levels than those with variants in other genes responsible for FH, a finding also observed in our patient cohort. Higher LDL-C levels throughout life, beginning in childhood, result in a cumulative LDL-C burden that inevitably leads to the development of atherosclerotic cardiovascular disease. In addition, the physiological consequences of the identified genetic variants can have an impact on the degree of LDLR dysfunction and the potential of pharmacological and gene therapies. Thus, loss of function variants have more impact on receptor activity and can benefit more from the gene therapy, while the variants leading to alterations in intracellular trafficking or increased protein degradation potentially can require other treatment strategies. Knowledge of the mechanism of LDLR dysfunction is also important for making an appropriate choice of contemporary lipid-lowing therapy. For example, gene-therapy or antibody-based PCSK9 ablation has no impact in case of homozygous LDLR loss, while potential AAV-based overexpression of LDLR can have no impact in case of genetically-determined PCSK9 gain of function. Thus, in light of the constantly developing new biological therapies the knowledge on the precise genetic background of the phenotype markedly contributes for the treatment efficacy.

An important group identified in our study is the group of disputed variants which included variants of unknown significance in FH-associated genes as well as the variants in other lipid-metabolism-related genes without clear prove of being causative for FH. Thus, *LDLRAP1* variants are considered as causative only for autosomal recessive hypercholesterolemia, its clinical and laboratory manifestations in heterozygous state in connection to FH phenotype is still under discussion. Additionally, the *APOE* variant p.Leu167del—the only known *APOE* variant associated with familial hypercholesterolemia—was identified in one patient [32]. This group of disputed variants may accommodate an important genetic potential for extension of FH-causative genes or new meaningful associations of variants in lipid-related genes and polygenic risk core (PRS) which can shed the light on the gene-elusive cases of FH-like phenotype with negative results of genetic studies. A more comprehensive investigation of the genetic basis of the disease across diverse populations is warranted, as the contribution of additional known or as-yet unidentified genes, polygenic risk factors, and secondary causes cannot be ruled out.

In our cohort of 30 patients, we identified genetic variants in genes associated with phenocopies of familial hypercholesterolemia. Notably, two patients carried variants of uncertain significance in the *ABCG5* and *ABCG8* genes, underscoring the molecular heterogeneity within the study group. These findings highlight a crucial clinical insight: the classical FH phenotype, as defined by DLCN criteria and biochemical parameters, is not entirely specific. This phenotype can be closely mimicked by other inherited dyslipidemias, most notably sitosterolemia and dysbetalipoproteinemia.

Sitosterolemia, an autosomal recessive disorder caused by pathogenic or likely pathogenic variants in *ABCG5* or *ABCG8*, is characterized by markedly elevated LDL-C levels, tendon xanthomas, and premature atherosclerosis, rendering it clinically indistinguishable from severe heterozygous or homozygous FH [33]. Similarly, dysbetalipoproteinemia (type III hyperlipoproteinemia), primarily driven by genetic variations in the *APOE* gene, may present with significant hypercholesterolemia, hypertriglyceridemia, and cutaneous xanthomas, which overlap extensively with the FH clinical spectrum [34]. The identification of these conditions within our clinically selected cohort demonstrates a critical limitation of traditional diagnostic algorithms: distinct genetic etiologies can converge into an identical clinical and laboratory phenotype. Consequently, expanded molecular screening using NGS panels that encompass a broader range of lipid metabolism genes—beyond the canonical *LDLR*, *APOB*, and *PCSK9*—is imperative. Such a comprehensive approach not only ensures an accurate definitive diagnosis but also has direct therapeutic implications. Standard FH therapies, such as statins, may be less effective in conditions like sitosterolemia, where specific dietary restrictions (elimination of plant sterols) and ezetimibe serve as the cornerstone of management. Therefore, implementing extended NGS panels enables personalized treatment strategies and helps avoid therapeutic pitfalls in patients presenting with FH phenocopies [35].

It should be noted that identifying familial hypercholesterolemia is complicated by its phenotypic overlap with familial combined hyperlipidemia, particularly regarding the presence of hypertriglyceridemia. Several studies have reported FH-causative variants in patients previously diagnosed with familial combined hyperlipidemia, prompting a recently proposed clinical classification for an FH subtype with hypertriglyceridemia, although the prevalence of hypertriglyceridemia in FH patients remains under 20%, which is significantly lower than in familial combined hyperlipidemia populations [36]. Of note, in our cohort, patients without identified causative FH variants exhibited significantly higher triglyceride levels compared to those with monogenic mutations, further underscoring the metabolic overlap between these two conditions and suggesting that genetically unresolved cases may present with a mixed dyslipidemic phenotype.

Additionally recent advances in whole-genome sequencing (WGS) have identified novel deep intronic *LDLR* variants that strongly co-segregate with the FH phenotype in affected families [37]. Screening these intronic regions is crucial for genetically negative FH patients, as certain variants can introduce premature stop codons. Similarly, variants in the *APOB* gene may exhibit incomplete penetrance in family cohorts. Consequently, WGS of the entire *APOB* gene is increasingly recommended, as pathogenic variants inducing autosomal dominant hypercholesterolemia are frequently located outside traditionally screened genomic regions [38].

Nevertheless, even extensive genetic screening does not always yield a definitive diagnosis. For example, in our cohort, one patient was found to have a maximum LDL-C level of 19.8 mmol/L (in the absence of secondary causes and elevated Lp(a) level), with only a modest reduction on combined lipid-lowering therapy; however, no pathogenic variants responsible for such marked LDL-C elevation were identified. This case underscores a critical point: the absence of a detectable monogenic variant does not equate to the absence of significant cardiovascular risk. Indeed, clinical outcomes differ significantly based on genetic status, even among patients with comparably severe LDL-C levels (e.g., ≥190 mg/dL). Individuals with a monogenic FH variant face a 22-fold increased risk of coronary artery disease compared to normocholesterolemic populations, whereas those with similar LDL-C elevations but no identified pathogenic variant exhibit only a 6-fold increase in risk [39]. Therefore, despite having nearly identical lipid profiles, monogenic FH patients carry a 2.5- to 3-fold higher risk of cardiovascular events than genetic-negative individuals [39,40]. This discrepancy in risk is driven by the cumulative lifelong cholesterol burden on the vasculature; in patients with a polygenic etiology or no identifiable monogenic pathology, cholesterol elevation is not necessarily congenital and is often modulated by age, lifestyle, and environmental factors, resulting in a shorter duration of vascular exposure. Nevertheless, despite lacking a confirmed monogenic variant, genetic-negative patients who present with severe LDL-C elevation and clinical signs of atherosclerosis still belong to a high-risk category for ASCVD and require aggressive lipid-lowering therapy. Therefore, the diagnostic workup must extend beyond standard gene panels, incorporating WGS to detect deep intronic and non-canonical variants, while maintaining a high index of clinical suspicion even in genetically unresolved cases.

As expected, genotype-positive FH cases were characterized by a significantly higher prevalence of tendon xanthomas with a similar frequency of corneal arcus compared to genotype-negative group. These findings underscore the fact that while certain clinical features are more indicative of the underlying monogenic etiology, the absence of such signs does not exclude the need for a comprehensive genetic diagnosis.

The subgroup of patients without identified pathogenic variants warrants particular attention. Among adult participants, 33.72% were genetically negative and 6.13% had disputed genetic findings. These patients still exhibited markedly elevated LDL-C levels. In spite of these LDL-C levels were lower compared to genotype-positive group, the frequency of premature coronary heart disease was even higher, although this difference did not reach statistical significance. In support of this observation, a higher frequency of cardiovascular events among relatives in the variant-negative group was also observed. One possible explanation may be linked to the fact that genetically negative patients were older at diagnosis (46 vs. 40 years) and had a greater burden of traditional cardiovascular risk factors. However, this group of patients may have had polygenic hypercholesterolemia or pathogenic variants in other genes not included in the analysis, potentially resulting in a different cardiovascular risk profile. Together these findings further emphasize that the absence of an identified pathogenic variant should not delay or preclude lipid-lowering treatment.

Analysis of the relationship between the clinical probability categories of FH according to the DLCN score and the results of genetic testing revealed a consistent increase in the proportion of genetically confirmed cases with increasing point scores. Thus, in the definite FH group (>8 points), the proportion of patients with pathogenic/likely pathogenic variants was 78.49%, while the proportion of genetically negative patients was 20.43%. Notably, in the possible FH group (3–5 points), a positive genetic result was obtained in half of the cases, demonstrating the insufficient sensitivity of the DLCN clinical criteria and highlighting the contemporary need for development of more accurate diagnostic tools for early detection of FH and further justifying the importance of genetic testing in all patients with suspected FH. These observations strongly support the implementation of systematic screening and the continuous improvement of diagnostic tools, as pathogenic variants in causative genes can be identified even in patients with low DLCN scores. With implementation of cascade genetic screening among relatives of patients with confirmed FH-associated variants such an approach would facilitate disease detection at a preclinical stage and enable timely initiation of preventive interventions, thereby reducing the burden of cardiovascular complications.

## 5. Conclusions

Using the genetic approach, we established that the patient cohort selected with clinical and laboratory data, as well as DLCN scores, includes both patients with FH and patients with sitosterolemia, dysbetalipoproteinemia, and other dyslipidemias. Variants in different genes responsible for lipid metabolism may present with identical phenotypic manifestations. Among patients with a DLCN score below 5 (3–5 points -possible FH), the genetic FH background was identified in 56.1% of cases. This finding underscores the high diagnostic yield of genetic testing across the entire clinical spectrum of FH and suggests that current clinical criteria may underestimate the likelihood of monogenic causes in patients with moderate DLCN scores. Although patients with a DLCN score > 6 points are more frequently referred for genetic testing, our results indicate that those with scores in the lower range also have a substantial chance of carrying a pathogenic variant. The presence of inconclusive genetic findings emphasizes the importance of studying the local genetic background, as population-specific variants may significantly influence the diagnostic yield of genetic testing in different regions.

In conclusion, genetic testing for FH should be considered not only in patients with high clinical probability but also in those with moderate DLCN scores. Further research into regional genetic landscapes and the functional characterization of VUS is essential to improve diagnostic accuracy and optimize clinical management.

## Figures and Tables

**Table 1:** Genes with identified variants in the investigated patient cohort.

Genes	Disease	Cite
*LDLR*, *APOB*, *PCSK9*, *LDLRAP1*	Familial hypercholesterolemia	[9]
FH phenocopy genes		
*ABCG5*, *ABCG8*	Sitosterolemia	[13]
*LIPA*	Deficiency of lysosomal acid lipase (Wolman disease and cholesteryl ester storage disease)/Coronary artery disease	[18,19]
FH candidate genes		
*APOE*	Familial dysbetalipoproteinemia	[9,14]
*STAP1*	Autosomal-dominant hypercholesterolemia	[20]
Other genes associated with lipid metabolism disorders		
*LPL*, *LMF1*, *GPIHBP1*, *APOA4*, *APOC3*	Hypertriglyceridemia	[21]
*APOA1*	Hypoalphalipoproteinemia	[22]
*CETP*	Hyperalphalipoproteinemia	[23]
*TNFRSF1B*, *LRP6*	Coronary artery disease	[24,25]

**Table 2:** Clinical and laboratory data of the patient group.

	All Subjects
Male (*n* = 261) ^a^	108 (41.38%)
Age, years (*n* = 261) ^b^	42 ± 13
BMI (kg/m^2^) (*n* = 259) ^c^	26.00 (23.30–29.65)
Tendon xanthomas (*n* = 261) ^a^	44 (16.86%)
Corneal arcus before age 45 (*n* = 261) ^a^	16 (6.13%)
Early coronary heart disease (*n* = 261) ^a^	44 (16.86%)
Atherosclerosis (*n* = 260) ^a^	8 (3.08%)
Cardiovascular events in relatives (*n* = 261) ^a^	132 (50.57%)
Elevated LDL-C levels in relatives (*n* = 261) ^a^	139 (53.26%)
Xanthomas in relatives (*n* = 261) ^a^	5 (1.92%)
Laboratory data	
Cholesterol, mmol/L (*n* = 261) ^c^	6.81 (4.99–8.55)
Triglycerides, mmol/L (*n* = 261) ^c^	1.10 (0.75–1.51)
HDL-C, mmol/L (*n* = 261) ^c^	1.32 (1.09–1.56)
Highest LDL-C level measured in life, mmol/L (*n* = 250) ^c^	7.57 (6.63–8.87)
LDL-C, mmol/L (*n* = 258) ^c^	4.83 (3.18–6.46)
Lp(a), mg/dL (*n* = 229) ^c^	23.00 (8.00–63.00)
Lipid-lowering therapy	
Atorvastatin (*n* = 261) ^a^	34 (13.03%)
Rosuvastatin (*n* = 261) ^a^	104 (39.85%)
Ezetimibe (*n* = 261) ^a^	72 (27.59%)
Fenofibrate (*n* = 261) ^a^	3 (1.15%)
Alirocumab (*n* = 261) ^a^	6 (2.30%)

Abbreviations: *n*, number of patients; BMI, body mass index; HDL-C, high-density lipoprotein; LDL-C, low-density lipoprotein; Lp(a), lipoprotein(a). ^a^ *n*, %; ^b^ Mean (standard deviation); ^c^ Mediana (interquartile range). Reference intervals: Cholesterol, mmol/L–3.10–5.20; Triglycerides, mmol/L–0.00–1.69; HDL-C, mmol/L–1.17–1.55; LDL-C, mmol/L–0.00–3.37; Lp(a), mg/dL–0.00–30.00.

**Table 3:** Comparison of patients with pathogenic/likely pathogenic FH gene variants and variant-negative patients.

	With P/LP Variants	Variant-Negative	*p* Value
Male	66 (42.04%); *n* = 157	38 (43.18%); *n* = 88	0.8933
Age when FH was diagnosed, years, mean ± SD	40 ± 13; *n* = 157	46 ± 14; *n* = 88	0.0017
BMI, kg/m^2^, median ± IQR	25.60 (22.75–29.10); *n* = 155	26.55 (23.64–29.90); *n* = 88	0.0915
Biochemical data			
Total cholesterol, mmol/L, median ± IQR	6.94 (5.19–8.90); *n* = 157	6.62 (4.83; 8.28); *n* = 88	0.1671
LDL-C, mmol/L, median ± IQR	4.92 (3.36–6.77); *n* = 156	4.62 (2.74–6.13); *n* = 87	0.0265
HiLDL, mmol/L, median ± IQR	7.83 (6.95–9.25); *n* = 151	7.08 (6.48–8.21); *n* = 83	0.0005
TG, mmol/L, median ± IQR	0.96 (0.72–1.41); *n* = 157	1.20 (0.93–1.54); *n* = 87	0.0239
HDL-C, mmol/L, median ± IQR	1.27 (1.06–1.54); *n* = 157	1.36 (1.16–1.61); *n* = 88	0.0337
LP(a), mg/dL, median ± IQR	21.00 (10.00–54.00); *n* = 139	22.50 (6.00–78.50); *n* = 76	0.9913
DLCN score components			
Tendon xanthoma	36 (22.93%); *n* = 157	7 (7.95%); *n* = 88	0.0028
Arcus cornealis before 45 years	12 (7.64%); *n* = 157	4 (4.55%); *n* = 88	0.4273
Early coronary heart disease	21 (13.38%); *n* = 157	20 (22.73%); *n* = 88	0.0743
Atherosclerosis	6 (3.85%); *n* = 156	2 (2.27%); *n* = 88	0.7146
High LDL levels in relatives	90 (57.32%); *n* = 157	42 (47.73%); *n* = 88	0.1816
Cardiovascular events in relatives	69 (43.95%); *n* = 157	53 (60.23%); *n* = 88	0.0029
Other parameters			
Xanthomas in relatives	3 (1.91%); *n* = 157	1 (1.14%); *n* = 88	>0.9999
Lipid-lowering therapy	84 (53.50%); *n* = 157	50 (57.47%); *n* = 87	0.5924

Abbreviations: *n*, number of patients; FH, familial hypercholesterolemia; BMI, body mass index; HDL-C, high-density lipoprotein; TG, Triglycerides; LDL-C, low-density lipoprotein; HiLDL, highest LDL-C level measured in life; Lp(a), lipoprotein(a); DLCN, Dutch Lipid Clinic Network; IQR, interquartile range. Reference intervals: Cholesterol, mmol/L—3.10–5.20; Triglycerides, mmol/L—0.00–1.69; HDL-C, mmol/L–1.17–1.55; LDL-C, mmol/L–0.00–3.37; Lp(a), mg/dL–0.00–30.00.

**Table 4:** LDL-C levels in all patients and in untreated patients according to genetic diagnosis.

Genetic Data		LDL-C Levels in All Patients, mmol/L	LDL-C Levels in Patients Without Therapy, mmol/L	*p* Value
Positive	median ± IQR	4.86 (3.41–6.70); *n* = 159	6.65 (5.85–7.95); *n* = 73	<0.0001
Disputed	median ± IQR	4.92 (3.36–6.10); *n* = 16	6.10 (5.93–6.71); *n* = 7	0.0626
Negative	median ± IQR	4.62 (2.74–6.13); *n* = 87	6.13 (4.76–7.17); *n* = 38	0.0002
Overall	median ± IQR	4.83 (3.18–6.46); *n* = 258	6.46 (5.69–7.86); *n* = 118	<0.0001

Abbreviations: *n*, number of patients; IQR, interquartile range; LDL-C, low-density lipoprotein; Positive, patients with pathogenic and likely pathogenic variants in FH genes; Disputed, patients with variants of uncertain clinical significance in FH genes and other lipid metabolism genes; Negative, patients without variants. Reference interval: LDL-C, mmol/L—0.00–3.37.

## Data Availability

Data is contained within the article or Supplementary Material.

## Supplementary Materials

The following supporting information can be downloaded at: https://www.mdpi.com/article/10.3390/jcdd13080384/s1, Table S1. Comparison of clinical and laboratory characteristics among patients of different age groups; Table S2. Clinical and laboratory parameters of patients with possible, probable, and definite FH; Table S3. Clinical and laboratory data of patients stratified into three groups according to genetic diagnosis; Table S4. Variants identified in FH-associated genes; Table S5. Variants identified in other lipid metabolism genes; Table S6. Clinical and laboratory parameters of patients receiving and not receiving lipid-lowering therapy at the time of enrolment in the database.

## Abbreviations

ACMG	American College of Medical Genetics and Genomics
APOB	Apolipoprotein B
ASCVD	Atherosclerotic cardiovascular disease
BMI	Body mass index
CADD	Combined Annotation Dependent Depletion
CHD	Coronary heart disease
D	Disputed variants (variant of uncertain significance in all genes, pathogenic, and likely pathogenic variants in other genes)
DLCN	Dutch Lipid Clinic Network
DNA	Deoxyribonucleic acid
EAS	European Atherosclerosis Society
FH	Familial hypercholesterolemia
gnomAD	Genome Aggregation Database
HDL-C	High-density lipoprotein
HeFH	Heterozygous familial hypercholesterolemia
HiLDL	Highest LDL-C level measured in life
HoFH	Homozygous familial hypercholesterolemia
IQR	Interquartile range
LDLR	Gene encoding low-density lipoprotein receptor
LDLRAP1	LDL receptor adaptor protein 1
LDL-C	Low-density lipoprotein cholesterol
LP	Likely pathogenic
Lp(a)	Lipoprotein(a)
MEDPED	Make Early Diagnoses to Prevent Early Deaths
n	Number of patients
N	No variants
NGS	Next-Generation Sequencing
P	Pathogenic
PCSK9	Proprotein convertase subtilisin/kexin type 9
TG	Triglycerides
VUS	Variants of uncertain significance
