# Supplementary material for: Genetic Spectrum of Familial Hypercholesterolemia in Russian North-West Registry: Focus on Correlation Between Clinical and Genetic Diagnosis
Source: J Cardiovasc Dev Dis. 2026 Aug 12;13(8):384. doi: 10.3390/jcdd13080384 (PMC13513221; doi:10.3390/jcdd13080384)
Supplement: Supplementary file 1 [file jcdd-13-00384-s001.zip › jcdd-4417945-supplementary.pdf]

Supplementary

Table S1. Comparison of clinical and laboratory characteristics among patients of different age groups.

	Age groups				
	**18 - 24**	**25 - 44**	**45 - 64**	**>65**	***p* Value**
Basic characteristics and biochemical data					
Male	13 (52.00%); *n*=25	63 (50.40%); *n*=125	30 (31.25%); *n*=96	2 (13.33%); *n*=15	0.0025
Age, mean±SD	21±2; *n*=25	35±5; *n*=125	53±6; *n*=96	70±5; *n*=15	<0.0001
Number of patients with variants in FH genes	20 (80.00%); *n*=25	82 (65.60%); *n*=125	51 (53.13%); *n*=96	8 (53.33%); *n*=15	0.0508
Total cholesterol, mmol/L, median± IQR	7.03 (5.21-8.45); *n*=25	7.08 (5.44-8.75); *n*=125	6.55 (4.82-8.35); *n*=96	5.32 (4.70-6.66); *n*=15	0.2099
LDL-C, mmol/L, median± IQR	5.15 (3.37-6.82); *n*=25	5.21 (3.65-6.54); *n*=125	4.62 (3.00-6.39); *n*=95	3.57 (2.72-4.65); *n*=14	0.1260
TG, mmol/L, median± IQR	0.90 (0.71-1.09); *n*=25	1.09 (0.72-1.43); *n*=125	1.24 (0.81-1.66); *n*=96	1.15 (0.91-1.32); *n*=15	0.0227
HDL-C, mmol/L, median± IQR	1.25 (1.08-1.68); *n*=25	1.35 (1.09-1.60); *n*=124	1.27 (1.11-1.55); *n*=96	1.15 (1.10-1.50); *n*=15	0.8166
HiLDL, mmol/L, median± IQR	6.90 (6.23-7.86); *n*=25	7.62 (6.60-8.98); *n*=122	7.62 (6.83-8.93); *n*=93	8.11 (7.59-9.16); *n*=10	0.0891
LP(a), mg/dL, median± IQR	17.00 (6.00-26.50); *n*=23	22.00 (8.00-56.25); *n*=110	26.00 (11.00-71.75); *n*=84	55.50 (12.25-94.00); *n*=12	<0.0001
DCLN score components					
Tendon xanthomas	1 (4.00%); *n*=25	18 (14.40%); *n*=125	20 (20.83%); *n*=96	5 (33.33%); *n*=15	0.0582
Arcus cornealis before 45 years	0 (0.00%); *n*=25	8 (6.40%); *n*=125	8 (8.33%); *n*=96	0 (0.00%); *n*=15	0.3289

Early coronary heart disease	0 (0.00%); *n*=25	18 (14.40%); *n*=125	23 (23.96%); *n*=96	3 (20.00%); *n*=15	0.0272
Atherosclerosis	0 (0.00%); *n*=25	1 (0.81%); *n*=124	7 (7.29%); *n*=96	0 (0.00%); *n*=15	0.0276
High LDL levels in relatives	18 (72.00%); *n*=25	69 (55.20%); *n*=125	41 (42.71%); *n*=96	11 (73.33%); *n*=15	0.0152
Cardiovascular events in relatives	12 (48.00%); *n*=25	61 (48.80%); *n*=125	53 (55.21%); *n*=96	6 (40.00%); *n*=15	0.6326
Other parameters					
Xanthomas in relatives	1 (4.00%); *n*=25	2 (1.60%); *n*=125	2 (2.08%); *n*=96	0 (0.00%); *n*=15	0.8130
Lipid-lowering therapy	13 (52.00%); *n*=25	57 (45.60%); *n*=125	60 (62.50%); *n*=96	13 (86.67%); *n*=15	0.0051
DLCN score, number of points					
3 – 5	10 (40.00%)	21 (16.80%)	7 (7.29%)	3 (20.00%)	0.0003
6 – 8	12 (48.00%)	62 (49.60%)	49 (51.04%)	4 (26.67%)	
>9	3 (12.00%)	42 (33.60%)	40 (41.67%)	8 (53.33%)	

Abbreviations: *n*, number of patients; FH, familial hypercholesterolemia; HDL-C, high-density lipoprotein; TG, Triglycerides; LDL-C, low-density lipoprotein; HiLDL, highest LDL-C level measured in life; Lp(a), lipoprotein(a); DLCN, Dutch Lipid Clinic Network; IQR, interquartile range.
Reference intervals: Cholesterol, mmol/L—3.10-5.20; Triglycerides, mmol/L—0.00-1.69; HDL-C, mmol/L—1.17-1.55; LDL-C, mmol/L—0.00-3.37; Lp(a), mg/dL—0.00-30.00.

Table S2. Clinical and laboratory parameters of patients with possible, probable, and definite FH.

	**DLCN score, number of points**			
	**3-5**	**6-8**	**≥9**	***p* Value**
Male	19 (46.34%); *n*=41	55 (43.31%); *n*=127	34 (36.56%); *n*=93	0.4720
Age, mean±SD	37±16; *n*=41	42±13; *n*=127	45±12; *n*=93	0.0021
Number of patients with variants in FH genes	23 (56.10%); *n*=41	64 (50.39%); *n*=127	74 (79.57%); *n*=93	<0.0001
Total cholesterol, mmol/L, median± IQR	6.81 (5.52-7.91); *n*=41	6.60 (4.77-8.36); *n*=127	7.05 (5.46-10.34); *n*=92	0.0171
LDL-C, mmol/L, median± IQR	4.70 (3.41-5.78); *n*=40	4.64 (2.81-6.26); *n*=126	5.39 (3.64-8.26); *n*=92	0.0018

HiLDL, mmol/L, median± IQR	6.21 (5.75-6.47); *n*=38	7.23 (6.64-7.82); *n*=121	9.16 (8.12-10.59); *n*=92	<0.0001
TG, mmol/L, median± IQR	1.10 (0.72-1.45); *n*=41	1.10 (0.75-1.52); *n*=126	1.10 (0.82-1.51); *n*=92	0.6665
HDL-C, mmol/L, median± IQR	1.40 (1.11-1.65); *n*=41	1.35 (1.14-1.61); *n*=127	1.20 (1.04-1.48); *n*=92	0.0099
LP(a), mg/dL, median± IQR	41.00 (13.00-80.00); *n*=35	20.00 (8.00-58.00); *n*=111	24.00 (8.00-54.00); *n*=83	0.2788
DCLN score components				
Tendon xanthomas	0 (0.00%); *n*=41	2 (1.57%); *n*=127	42 (45.16%); *n*=93	<0.0001
Arcus cornealis before 45 years	0 (0.00%); *n*=41	0 (0.00%); *n*=127	16 (17.20%); *n*=93	<0.0001
Early coronary heart disease	1 (2.44%); *n*=41	27 (21.26%); *n*=127	16 (17.20%); *n*=93	0.0198
Atherosclerosis	1 (2.44%); *n*=41	3 (2.36%); *n*=127	4 (4.35%); *n*=93	0.6896
High LDL levels in relatives	20 (48.78%); *n*=41	70 (55.12%); *n*=127	49 (52.69%); *n*=93	0.7715
Cardiovascular events in relatives	15 (36.59%); *n*=41	74 (58.27%); *n*=127	43 (46.24%); *n*=93	0.0315
Other parameters				
Xanthomas in relatives	1 (2.44%); *n*=41	3 (2.36%); *n*=127	1 (1.08%); *n*=93	0.7618
Lipid-lowering therapy	16 (39.02%); *n*=41	76 (59.84%); *n*=127	51 (54.84%); *n*=93	0.0664

Abbreviations: *n*, number of patients; FH, familial hypercholesterolemia; HDL-C, high-density lipoprotein; TG, Triglycerides; LDL-C, low-density lipoprotein; HiLDL, highest LDL-C level measured in life; Lp(a), lipoprotein(a); DLCN, Dutch Lipid Clinic Network; IQR, interquartile range.

Reference intervals: Cholesterol, mmol/L—3.10-5.20; Triglycerides, mmol/L—0.00-1.69; HDL-C, mmol/L—1.17-1.55; LDL-C, mmol/L—0.00-3.37; Lp(a), mg/dL—0.00-30.00.

Table S3. Clinical and laboratory data of patients stratified into three groups according to genetic diagnosis.

	**With P/LP Variants**	**With disputed variants**	**Variant-negative**
Male	66 (42.04%); *n*=157	4 (25.00%); *n*=16	38 (43.18%); *n*=88
Age when FH was diagnosed, years, mean±SD	40±13; *n*=157	43±14; *n*=16	46±14; *n*=88
BMI, kg/m2, median± IQR	25.60 (22.75-29.10); *n*=155	27.15 (25.57-30.15); *n*=16	26.55 (23.64-29.90); *n*=88
Biochemical data			

Total cholesterol, mmol/L, median± IQR	6.94 (5.19-8.90); *n*=157	6.53 (4.65-8.28); *n*=16	6.62 (4.83-8.28); *n*=88
LDL-C, mmol/L, median± IQR	4.92 (3.36-6.77); *n*=156	4.62 (2.60-6.05); *n*=15	4.62 (2.74-6.13); *n*=87
HiLDL, mmol/L, median± IQR	7.83 (6.95-9.25); *n*=151	6.86 (6.30-7.52); *n*=16	7.08 (6.48-8.21); *n*=83
TG, mmol/L, median± IQR	0.96 (0.72-1.41); *n*=157	1.50 (1.10-2.19); *n*=16	1.20 (0.93-1.54); *n*=87
HDL-C, mmol/L, median± IQR	1.27 (1.06-1.54); *n*=157	1.35 (1.05-1.58); *n*=16	1.36 (1.16-1.61); *n*=88
LP(a), mg/dL, median± IQR	21.00 (10.00-54.00); *n*=139	33.50 (9.00-53.50); *n*=14	22.50 (6.00-78.50); *n*=76
DLCN score components			
Tendon xanthoma	36 (22.93%); *n*=157	1 (6.25%); *n*=16	7 (7.95%); *n*=88
Arcus cornealis before 45 years	12 (7.64%); *n*=157	0 (0.00%); *n*=16	4 (4.55%); *n*=88
Early coronary heart disease	21 (13.38%); *n*=157	3 (18.75%); *n*=16	20 (22.73%); *n*=88
Atherosclerosis	6 (3.85%); *n*=156	0 (0.00%); *n*=16	2 (2.27%); *n*=88
High LDL levels in relatives	90 (57.32%); *n*=157	8 (50.00%); *n*=16	42 (47.73%); *n*=88
Cardiovascular events in relatives	69 (43.95%); *n*=157	10 (62.50%); *n*=16	53 (60.23%); *n*=88
Other parameters			
Xanthomas in relatives	3 (1.91%); *n*=157	1 (6.25%); *n*=16	1 (1.14%); *n*=88
Lipid-lowering therapy	84 (53.50%); *n*=157	9 (56.25%); *n*=16	50 (57.47%); *n*=87

Abbreviations: *n*, number of patients; FH, familial hypercholesterolemia; BMI, body mass index; HDL-C, high-density lipoprotein; TG, Triglycerides; LDL-C, low-density lipoprotein; HiLDL, highest LDL-C level measured in life; Lp(a), lipoprotein(a); DLCN, Dutch Lipid Clinic Network; IQR, interquartile range.

Reference intervals: Cholesterol, mmol/L—3.10-5.20; Triglycerides, mmol/L—0.00-1.69; HDL-C, mmol/L—1.17-1.55; LDL-C, mmol/L—0.00-3.37; Lp(a), mg/dL—0.00-30.00.

Table S4. Variants identified in FH-associated genes.

Gene	dbSNP	Chromosomal position	cDNA position	Genetic variant at protein level	ACMG Classification	Type of Variant	Frequency of Variant in the population	CADD	Number of patients
LDLR	rs121908038	chr19:g.11113293T>A	NM_000527.5:c.1202T>A	p.Leu401His	Pathogenic	Missense	0.000248%	27.0	21
LDLR	rs137929307	chr19:g.11116928G>A	NM_000527.5:c.1775G>A	p.Gly592Glu	Pathogenic	Missense	0.004647%	25.1	12
LDLR	rs750518671	chr19:g.11128085G>A	NM_000527.5:c.2389G>A	p.Val797Met	Pathogenic	Missense	0.00043%	25.4	5
LDLR	rs769737896	chr19:g.11110759C>T	NM_000527.5:c.1048C>T	p.Arg350Ter	Pathogenic	Nonsense	0.0008059%	36.0	4
LDLR	rs1600727337	chr19:g.11113558del	NM_000527.5:c.1382del	p.Gly461AlafsTer46	Pathogenic	Frameshift, deletion	0.000062%	10.4	2
LDLR	rs773618064	chr19:g.11129534T>TG	NM_000527.5:c.2416dup	p.Val806GlyfsTer11	Pathogenic	Frameshift, duplication	0.001177%	24.5	3
LDLR	rs879254492	chr19:g.11105267T>G	NM_000527.5:c.361T>G	p.Cys121Gly	Pathogenic	Missense	0.000062%	25.5	2
LDLR	rs137943601	chr19:g.11113313G>A	NM_000527.5:c.1222G>A	p.Glu408Lys	Pathogenic	Missense	0.0002479%	27.6	1
LDLR	rs121908027	chr19:g.11105560_11105562del	NM_000527.5:c.654_656del	p.Gly219del	Pathogenic	Missense	0.00188%	9.98	2
LDLR	rs570942190	chr19:g.11224013C>T	NM_000527.5:c.1246C>T	p.Arg416Trp	Pathogenic	Missense	0.00239%	Not available	2
LDLR	rs773328511	chr19:g.11106680C>A	NM_000527.5:c.810C>A	p.Cys270Ter	Pathogenic	Nonsense	0.00006195%	26.1	3
LDLR	rs144172724	chr19:g.11102774G>T	NM_000527.5:c.301G>T	p.Glu101Ter	Pathogenic	Nonsense	Not available	Not available	2
LDLR	rs879254866	chr19:g.11113419G>A	NM_000527.5:c.1328G>A	p.Trp443Ter	Pathogenic	Nonsense	0.00006196%	43.0	2

LDLR	rs879254474	chr19:g.11105221CCCCAAGACGTGCT>C	NM_000527.5:c.320_332del	p.Lys107ArgfsTer95	Pathogenic	Frameshift, deletion	Not available	Not available	1
LDLR	rs756613387	chr19:g.11105572C>A	NM_000527.5:c.666C>A	p.Cys222Ter	Pathogenic	Nonsense	Not available	Not available	1
LDLR	rs730882096	chr19:g.11110738G>A	NM_000527.5:c.1027G>A	p.Gly343Ser	Pathogenic	Missense	0.003408%	28.7	2
LDLR	rs875989936	chr19:g.11120201T>C	NM_000527.5:c.1955T>C	p.Met652Thr	Pathogenic	Missense	0.0001239%	24.6	1
LDLR	rs879254667	chr19:g.11106631A>C	NM_000527.5:c.761A>C	p.Gln254Pro	Pathogenic	Missense	0.0006195%	26.9	2
LDLR		chr19	NM_000527.5:c.(940+1_941-1)_(1845+1_1846-1)del		Pathogenic	Deletion	Not available	Not available	2
LDLR	rs1555807335	chr19:g.11120369G>A	NM_000527.5:c.1988-1G>A		Pathogenic	Splice-site mutations	Not available	35.0	1
LDLR	rs1600743301	chr19:g.11120379GGT>G	NM_000527.5:c.2001_2002delTG	p.Cys667Ter	Pathogenic	Nonsense	Not available	Not available	1
LDLR		chr19	NM_000527.5:c.(313+1_314-1)_(940+1_941-1)del		Pathogenic	Deletion	Not available	Not available	1
LDLR		chr19	NM_000527.5:c.(?_314)_(940_?)del		Pathogenic	Deletion	Not available	Not available	1
LDLR	rs875989906	chr19:g.11105567G>A	NM_000527.5:c.661G>A	p.Asp221Asn	Pathogenic	Missense	0.0005585%	26.40	1
LDLR		chr19:g.11107440G>T	NM_000527.5:c.866G>T	p.Cys289Phe	Pathogenic	Missense	Not available	Not available	1
LDLR	rs879254566	chr19:g.11105440T>G	NM_000527.5:c.534T>G	p.Asp178Glu	Pathogenic	Missense	Not available	Not available	1
LDLR	rs879254652	chr19:g.11106564G>T	NM_000527.5:c.695-1G>T		Pathogenic	Splice-site mutations	Not available	35.0	1
LDLR	rs879254803	chr19:g.11111588T>C	NM_000527.5:c.1135T>C	p.Cys379Arg	Pathogenic	Missense	0.00006197%	29.1	1

LDLR	rs387906304	chr19:g.11107497AACCCATC>A	NM_000527.5:c.925_931del	p.Pro309LysfsTer59	Pathogenic	Frameshift, deletion	0.0009324%	27.4	1
LDLR	rs139043155	chr19:g.11106668T>A	NM_000527.5:c.798T>A	p.Asp266Glu	Pathogenic	Missense	0.003222%	15.7	1
LDLR	rs879255000	chr19:g.11116882T>C	NM_000527.5:c.1729T>C	p.Trp577Arg	Pathogenic	Missense	0.00006196%	27.0	1
LDLR	rs201102492	chr19:g.11116937G>A	NM_000527.5:c.1784G>A	p.Arg595Gln	Pathogenic	Missense	0.001611%	28.6	1
LDLR	rs730882109	chr19:g.11116900C>T	NM_000527.5:c.1747C>T	p.His583Tyr	Pathogenic	Missense	0.002788%	24.7	1
LDLR	rs1555808044	chr19:g.11123213_11123217dup	NM_000527.5:c.2180_2184dup	p.Leu729ValfsTer3	Pathogenic	Frameshift, duplication	Not available	Not available	1
LDLR	rs28942084	chr19:g.11120436C>T	NM_000527.5:c.2054C>T	p.Pro685Leu	Pathogenic	Missense	0.00347%	24.9	1
LDLR		chr19:g.11116859A>T	NM_000527.5:c.1706A>T	p.Asp569Val	Pathogenic	Missense	Not available	Not available	1
LDLR		chr19:g.11113641T>A	NM_000527.5:c.1465T>A	p.Tyr489Asn	Pathogenic	Missense	Not available	Not available	1
LDLR	rs28942078	chr19:g.11113376G>A	NM_000527.5:c.1285G>A	p.Val429Met	Pathogenic	Missense	0.001301%	25.3	1
LDLR	rs879254783	chr19:g.11111525T>C	NM_000527.5:c.1072T>C	p.Cys358Arg	Pathogenic	Missense	Not available	27.7	1
LDLR	rs879255175	chr19:g.11123345G>A	NM_000527.5:c.2311+1G>A		Pathogenic	Splice-site mutations	0.00006197%	33.0	1
LDLR	rs879255003	chr19:g.11116889A>T	NM_000527.5:c.1736A>T	p.Asp579Val	Pathogenic	Missense	Not available	25.8	1
LDLR		chr19:g.11116940_11116963del	NM_000527.5:c.1787_1810del	p.Lys596_Lys603del	Pathogenic	Deletion	Not available	Not available	1
LDLR	rs144172724	chr19:g.11102774G>A	NM_000527.5:c.301G>A	p.Glu101Lys	Pathogenic	Missense	0.002851%	25.20	2

LDLR	rs761954844	chr19:g.11110697G>A	NM_000527.5:c.986G>A	p.Cys329Tyr	Likely Pathogenic	Missense	0.0006197%	25.3	6
LDLR		chr19:g.11116203A>T	NM_000527.5:c.1696A>T	p.Ile566Phe	Likely Pathogenic	Missense	Not available	26.3	3
LDLR	rs1301458707	chr19:g.11116885G>T	NM_000527.5:c.1732G>T	p.Val578Phe	Likely Pathogenic	Missense	Not available	27.0	3
LDLR	rs879254671	chr19:g.11106638C>A	NM_000527.5:c.768C>A	p.Asp256Glu	Likely Pathogenic	Missense	0.0001239%	22.9	1
LDLR		chr19:g.11107517_11107520del	NM_000527.5:c.940+3_940+6del; delGTGA		Likely Pathogenic	Deletion	Not available	Not available	1
LDLR		chr19:g.11116920dup	NM_000527.5:c.1767dup	p.Val590CysfsTer13	Likely Pathogenic	Frameshift, duplication	Not available	Not available	1
LDLR		chr19:g.11113410del	NM_000527.5:c.1319del	p.Arg440LysfsTer11	Likely Pathogenic	Frameshift, deletion	Not available	Not available	1
LDLR		chr19:g.11129597A>T	NM_000527.5:c.2474A>T	p.Asn825Ile	Likely Pathogenic	Missense	Not available	Not available	1
LDLR		chr19:g.11116162_11116179del	NM_000527.5:c.1655_1672del;	p.Ile552_Glu558delinsLys	Likely Pathogenic	Deletion	Not available	Not available	2
LDLR	rs879254850	chr19:g.11113365A>T	NM_000527.5:c.1274A>T	p.Asn425Ile	Likely Pathogenic	Missense	Not available	23.6	2
LDLR	rs753707206	chr19:g.11116954G>T	NM_000527.5:c.1801G>T	p.Asp601Tyr	Likely Pathogenic	Missense	Not available	Not available	1
LDLR		chr19:g.11120224C>CA	NM_000527.5:c.1979dup	p.Pro661AlafsTer8	Likely Pathogenic	Frameshift, duplication	Not available	Not available	1
LDLR	rs879255010	chr19:g.11116903T>C	NM_000527.5:c.1750T>C	p.Ser584Pro	Likely Pathogenic	Missense	Not available	Not available	1

LDLR		chr19:g.11120214C>A	NM_000527.5:c.1968C>A	p.His656Gln	Likely Pathogenic	Missense	Not available	Not available	1
LDLR	rs5742904	chr19:g.11105261G>GTTCC	NM_000527.5:c.355_356insTTCC	p.Gly119ValfsTer12	Likely Pathogenic	Frameshift, insertion	Not available	Not available	2
LDLR		chr19:g.11120476C>A	NM_000527.5:c.2094C>A	p.Cys698Ter	Likely Pathogenic	Nonsense	Not available	Not available	1
LDLR	rs879255051	chr19:g.11120091G>C	NM_000527.5:c.1846-1G>C		Likely Pathogenic	Splice-site mutations	Not available	Not available	1
LDLR		chr19:g.11110649C>G	NM_000527.5:c.941-3C>G		Likely Pathogenic	Splice-site mutations	Not available	13.9	1
LDLR	rs879254528	chr19:g.11105350T>G	NM_000527.5:c.444T>G	p.Cys148Trp	Likely Pathogenic	Missense	Not available	16.85	1
LDLR	rs377437226	chr19:g.11129669C>A	NM_000527.5:c.2546C>A	p.Ser849Ter	Likely Pathogenic	Nonsense	0.000372%	45.0	1
LDLR	rs879255086	chr19:g.11120204T>G	NM_000527.5:c.1958T>G	p.Val653Gly	Likely Pathogenic	Missense	Not available	25.8	1
LDLR		chr19:g.11105571G>GGGC	NM_000527.5:c.665_666insGGC	p.Cys222delinsTrpArg	Likely Pathogenic	Insertion	Not available	Not available	1
LDLR	rs879254492	chr19:g.11105267T>C	NM_000527.5:c.361T>C	p.Cys121Arg	Likely Pathogenic	Missense	Not available	Not available	1
LDLR		chr19:g.11113368T>G	NM_000527.5:c.1277T>G	p.Leu426Arg	Likely Pathogenic	Missense	Not available	Not available	1
LDLR		chr19:g.11105562GCCCCGACT>G	NM_000527.5:c.657_664del	p.Pro220GlnfsTer5	Likely Pathogenic	Frameshift, deletion	Not available	Not available	1
LDLR	rs146200173	chr19:g.11113292C>T	NM_000527.5:c.1201C>T	p.Leu401Phe	Likely Pathogenic	Missense	0.00006199%	25.4	1

LDLR		chr19:g.11116879T>G	NM_000527.5:c.1726T>G	p.Tyr576Asp	Likely Pathogenic	Missense	Not available	27.1	1
LDLR	rs577934998	chr19:g.11105570T>C	NM_000527.5:c.664T>C	p.Cys222Arg	Likely Pathogenic	Missense	0.0001242%	26.6	1
LDLR		chr19:g.11105387AT>A	NM_000527.5:c.482del; delT	p.Ile161ThrfsTer45	Likely Pathogenic	Frameshift, deletion	Not available	Not available	1
LDLR		chr19:g.11113635A>G	NM_000527.5:c.1459A>G	p.Asn487Asp	Likely Pathogenic	Missense	Not available	24.0	1
LDLR		chr19:g.11100283A>G	NM_000527.5:c.128A>G	p.Lys43Arg	Variant of Uncertain Significance	Missense	Not available	22.6	1
LDLR	rs730882111	chr19:g.11120200A>G	NM_000527.5:c.1954A>G	p.Met652Val	Variant of Uncertain Significance	Missense	0.0001239%	19.7	1
LDLR	rs879254889	chr19:g.11113570A>G	NM_000527.5:c.1394A>G	p.Tyr465Cys	Variant of Uncertain Significance	Missense	Not available	24.9	1
APOB	rs5742904	chr2:g.21006288C>T	NM_000384.3:c.10580G>A	p.Arg3527Gln	Pathogenic	Missense	0.04207%	26.7	19
APOB		chr2:g.21007707C>T	NM_000384.3:c.9161G>A	p.Gly3054Glu	Variant of Uncertain Significance	Missense	Not available	24.1	1
PCSK9	rs374603772	chr1:g.55058630C>T	NM_174936.4:c.1486C>T	p.Arg496Trp	Variant of Uncertain Significance	Missense	0.004785%	23.3	2
LDLRAP1	rs781585299	chr1:g.25563140dup	NM_015627.3:c.603dup	p.Ser202LeufsTer19	Pathogenic	Frameshift, duplication	0.003355%	24.1	1

LDLRAP1	rs867506518	chr1:g.25557244T>C	NM_015627.3:c.436T>C	p.Phe146Leu	Variant of Uncertain Significance	Missense	0.0032%	Not available	1

Table S5. Variants identified in other lipid metabolism genes.

Gene	dbSNP	Chromosomal position	cDNA position	Genetic variant at protein level	ACMG Classification	Type of Variant	Frequency of Variant in the population	CADD	Number of patients
APOE	rs515726148	chr19:g.44908792-44908794	NM_000041.4:c.500_502delCTC	p.Leu167del	Pathogenic	Deletion	0.004119%	16.2	1
APOE	rs28931579	chr19:g.44909236A>C	NM_000041.4:c.940A>C	p.Ser314Arg	Pathogenic	Missense	0.005574%	13.3	1
LIPA	rs116928232	chr10:g.89222511C>T	NM_000235.4:c.894G>A	p.Gln298=	Pathogenic	Splice-site mutations	0.1111%	24.7	1
LPL	rs773407951	chr8:g.19955883A>G	NM_000237.3:c.818A>G	p.His273Arg	Likely Pathogenic	Missense	0.0006195%	26.1	1
TNFRSF1B	rs1208863994	chr1:g.12206739G>T	NM_001066.3:c.1106-1G>T		Likely Pathogenic	Splice-site mutations	Not available	Not available	1
ABCG5	rs150401285	chr2:g.43813202A>G	NM_022436.3:c.1870T>C	p.Phe624Leu	Variant of Uncertain Significance	Missense	0.07486%	24.1	1
ABCG8	rs776335488	chr2:g.43875362T>C	NM_022437.3:c.1705T>C	p.Ser569Pro	Variant of Uncertain Significance	Missense	0.0003098%	22.9	1

APOA1	COSV52637012	chr11:g.116836035C>A	NM_000039.3:c.577G>T	p.Glu193Ter	Variant of Uncertain Significance	Nonsense	Not available	33.0	1
APOA1	rs777476426	chr11:g.116836250A>G	NM_000039.3:c.362T>C	p.Val121Ala	Variant of Uncertain Significance	Missense	0.00006195%	25.6	1
APOA4	rs781747963	chr11:g.116821148G>A	NM_000482.4:c.910C>T	p.Arg304Ter	Variant of Uncertain Significance	Missense	0.006817%	37.0	2
APOE		chr19:g.44909171G>T	NM_000041.4:c.875G>T	p.Arg292Leu	Variant of Uncertain Significance	Missense	0.0001875%	22.8	2
APOE	rs267606664	chr19:g.44908730G>A	NM_000041.4:c.434G>A	p.Gly145Asp	Variant of Uncertain Significance	Missense	0.01673%	24.5	1
CETP	rs147566657	chr16:g.56973406C>T	NM_000078.3:c.826C>T	p.Arg276Cys	Variant of Uncertain Significance	Missense	0.0009293%	24.8	1
CETP	rs753876598	chr16:g.56963056del	NM_000078.3:c.165del	p.Ser56AlafsTer11	Variant of Uncertain Significance	Frameshift, deletion	0.001301%	13.5	1
GPIHBP1	rs145844329	chr8:g.143215486G>C	NM_178172.6:c.523G>C	p.Gly175Arg	Variant of Uncertain Significance	Missense	0.073180%	15.1	1
LIPA	rs756878837	chr10:g.89225151C>T	NM_000235.4:c.616G>A	p.Val206Ile	Variant of Uncertain Significance	Missense	0.0005576%	0.001	1

LMF1	rs928471580	chr16:g.879617C>T	NM_022773.4:c.850G>A	p.Gly284Ser	Variant of Uncertain Significance	Missense	0.004959%	23.4	1
LRP6		chr12:g.12135237C>G	NM_002336.3:c.3671G>C	p.Gly1224Ala	Variant of Uncertain Significance	Missense	Not available	25.5	1
LRP6	rs779010191	chr12:g.12121368T>G	NM_002336.3:c.4600A>C	p.Thr1534Pro	Variant of Uncertain Significance	Missense	0.001611%	27.7	1
LRP6	rs144175121	chr12:g.12125309G>A	NM_002336.3:c.4436C>T	p.Thr1479Ile	Variant of Uncertain Significance	Missense	0.06419%	23.2	1
STAP1	rs199787258	chr4:g.67581467C>T	NM_012108.4:c.526C>T	p.Pro176Ser	Variant of Uncertain Significance	Missense	0.0404%	25.1	3
STAP1	rs781246980	chr4:g.67571138G>A	NM_012108.4:c.175G>A	p.Asp59Asn	Variant of Uncertain Significance	Missense	0.001121%	22.8	1
TNFRSF1B	rs989561625	chr1:g.12202168T>C	NM_001066.3:c.1102T>C	p.Ser368Pro	Variant of Uncertain Significance	Missense	0.0005175%	19.6	1

Table S6. Clinical and laboratory parameters of patients receiving and not receiving lipid-lowering therapy at the time of enrolment in the database.

	**Lipid-lowering therapy**		
	**Not receiving lipid-lowering therapy**	**Receiving lipid-lowering therapy**	**p Value**
Male	44 (37.29%); *n*=118	64 (44.76%); *n*=143	0.2562
Age when FH was diagnosed, years, mean±SD	40±12; *n*=118	45±14; *n*=143	0.0022
BMI, kg/m2, median± IQR	25.60 (22.80-29.03); *n*=117	26.39 (23.75-29.70); *n*=142	0.1653
	Biochemical data		
Total cholesterol, mmol/L, median± IQR	8.32 (7.29-9.49); *n*=162	5.43 (4.16-6.71); *n*=147	<0.0001
LDL-C, mmol/L, median± IQR	6.38 (5.40-7.46); *n*=162	3.47 (2.54-4.62); *n*=144	<0.0001
TG, mmol/L, median± IQR	0.99 (0.76-1.37); *n*=161	1.02 (0.74-1.43); *n*=147	0.1483
HDL-C, mmol/L, median± IQR	1.36 (1.15-1.58); *n*=162	1.28 (1.08-1.56); *n*=147	0.2594
HiLDL, mmol/L, median± IQR	7.40 (6.50-8.60); *n*=161	7.51 (6.64-8.70); *n*=137	0.8273
LP(a), mg/dL, median± IQR	19.50 (9.00-51.00); *n*=142	25.00 (8.00-80.00); *n*=123	0.1658
	DLCN score components		
Tendon xanthomas	17 (14.41%); *n*=118	27 (18.88%); *n*=143	0.4069
Arcus cornealis before 45 years	4 (3.39%); *n*=118	12 (8.39%); *n*=143	0.1213
Early coronary heart disease	5 (4.24%); *n*=118	39 (27.27%); *n*=143	<0.0001
Atherosclerosis	2 (1.69%); *n*=118	6 (4.23%); *n*=142	0.2984
High LDL levels in relatives	71 (60.17%); *n*=118	68 (47.55%); *n*=143	0.0467
Cardiovascular events in relatives	59 (50.00%); *n*=118	73 (51.05%); *n*=143	0.9014
Xanthomas in relatives	3 (2.54%); *n*=118	2 (1.40%); *n*=143	0.6608
Variant in the FH genes	74 (62.71%); *n*=118	87 (60.84%); *n*=143	0.7987

Abbreviations: *n*, number of patients; BMI, body mass index; FH, familial hypercholesterolemia; HDL-C, high-density lipoprotein; TG, Triglycerides; LDL-C, low-density lipoprotein; HiLDL, highest LDL-C level measured in life; Lp(a), lipoprotein(a); DLCN, Dutch Lipid Clinic Network; IQR, interquartile range.

Reference intervals: Cholesterol, mmol/L—3.10-5.20; Triglycerides, mmol/L—0.00-1.69; HDL-C, mmol/L—1.17-1.55; LDL-C, mmol/L—0.00-3.37; Lp(a), mg/dL—0.00-30.00.
